# Supplementary material for: Inhibitory Activity of Saussurea costus Extract against Bacteria, Candida, Herpes, and SARS-CoV-2
Source: Plants (Basel). 2023 Jan 19;12(3):460. doi: 10.3390/plants12030460 (PMC9920761; doi:10.3390/plants12030460)
Supplement: Supplementary file 1 [file plants-12-00460-s001.zip › plants-2104181-supplementary.pdf]

Supplementary file

Table S1. GC-MS analysis conditions

Integration	Gas Chromatography real time analysis software
Detector	GC/MS QP2012 Ultra
Column	Rtx-5ms
Column length	30 m
Inside column diameter	0.25 mm
Film thickness	0.25 µm
Column temperature program	60 °C increased at 10°C to 300°C, held for 10 min
Detector temperature	200 °C
Injector temperature	250 °C
Carrier gas, inlet pressure	helium ,1.6ml/min
Split ratio	0.3 ml/min
Injection volume	0.1 µl

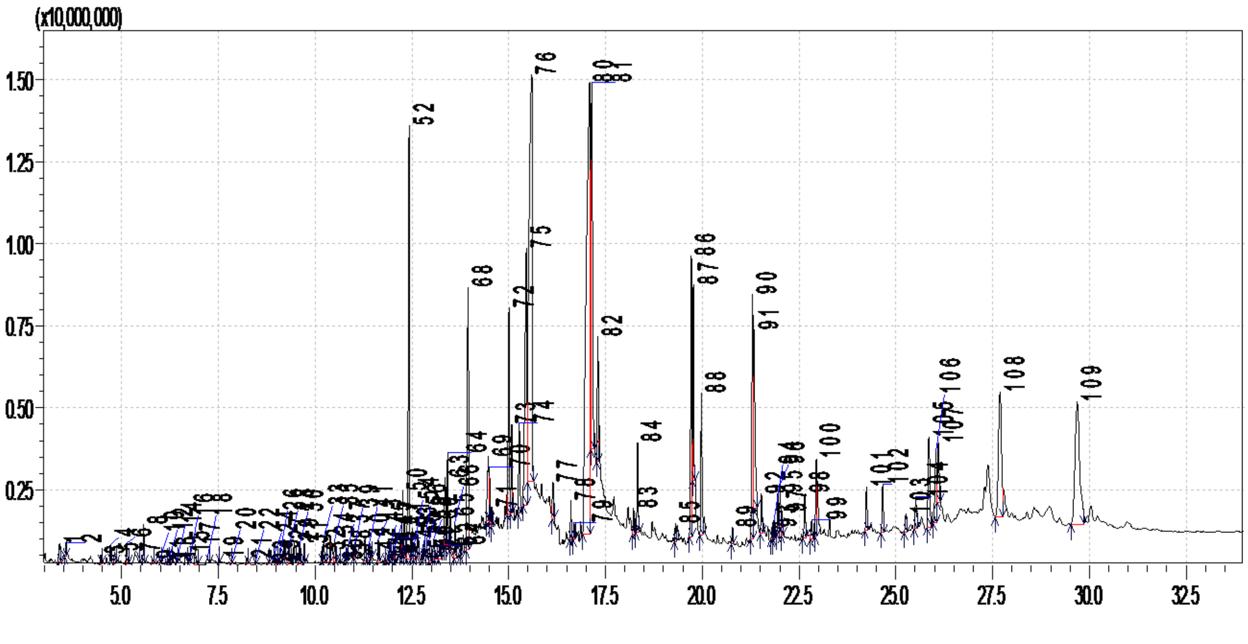

Figure S1. GC-MS chromatograms of acetic acid extract of *Saussurea costus*.

Table S2. Phytochemical in acetic acid extraction of *Saussurea costus* roots.

ID#	Name	Area%
1	Pentanoic acid, 4-oxo-	0.18
2	Di(1,2,5-oxadiazolo)[3,4-b:3,4-E]pyrazine, 4,8-diacetyl-	0.04
3	2-Caren-10-al	0.03
4	4-Terpinenyl acetate	0.02
5	Phenol, 2,6-dimethoxy-	0.06
6	Phenol, 2-methoxy-4-(2-propenyl)-, acetate	0.01
7	Methyleugenol	0.03
8	2-Butanone, 4-(2,6,6-trimethyl-2-cyclohexen-1-yl)-	0.16
9	.alpha.-Ionone	0.17
10	Bicyclo[7.2.0]undec-4-ene, 4,11,11-trimethyl-8-methylene-	0.19
11	5,9-Undecadien-2-one, 6,10-dimethyl-	0.15
12	trans-.beta.-Ionone	0.10
13	Benzene, 1-(1,5-dimethyl-4-hexenyl)-4-methyl-	0.24
14	2-Isopropenyl-4a,8-dimethyl-1,2,3,4,4a,5,6,8a-octahydronaphthalene	0.23
15	.beta.-Humulene	0.20
16	.beta.-Bisabolene	0.03
17	Caryophyllene	0.06
18	Benzene, 1,2,3-trimethoxy-5-(2-propenyl)-	0.06
19	3,7-Cyclodecadiene-1-methanol, .alpha.,.alpha.,4,8-tetramethyl-, [s-(Z,Z)]	0.09
20	Cyclohexane, 1-ethenyl-1-methyl-2,4-bis(1-methylethenyl)-, [1S-(1.alpha.,2.beta.,4.beta.)]-	0.24
21	Phenol, 2,6-dimethoxy-4-(2-propenyl)-	0.13
22	Caryophyllene oxide	0.07
23	1-Heptadecene	0.19
24	(-)-Isolongifolol, acetate	0.03

25	Benzaldehyde, 4-hydroxy-3,5-dimethoxy-	0.09
26	Cyclopenta[1,3]cyclopropa[1,2]cyclohepten-3(3aH)-one, 1,2,3b,6,7,8-hexahydro-6,6-dimethyl-	0.04
27	2-Naphthalenemethanol, 1,2,3,4,4a,5,6,7-octahydro-.alpha.,.alpha.,4a,8-tetramethyl-, (2R-cis)-	0.02
28	Tetracyclo[6.3.2.0(2,5).0(1,8)]tridecan-9-ol, 4,4-dimethyl-	0.05
29	2-Naphthalenemethanol, decahydro-.alpha.,.alpha.,4a-trimethyl-8-methylene-, [2R-(2.alpha.,4a.alpha.,8a.beta.)]-	0.13
30	Bicyclo[5.3.0]decane, 2-methylene-5-(1-methylvinyl)-8-methyl-	0.47
31	Bicyclo[10.6.0]octadeca-1(12),15-diene	0.29
32	Naphthalene, decahydro-4a-methyl-1-methylene-7-(1-methylethenyl)-, [4aR-(4a.alpha.,7.alpha.,8a.beta.)]-	0.13
33	Oxacyclododeca-6,9-dien-2-one, 7-methyl-, (Z,E)-	0.25
34	6-Isopropenyl-4,8a-dimethyl-1,2,3,5,6,7,8,8a-octahydro-naphthalen-2-ol	0.06
35	1-Isopropenyl-3,3-dimethyl-5-(3-methyl-1-oxo-2-butenyl)cyclopentane	0.03
36	1-Heptatriacotanol	0.13
37	9H-Fluorene, 9-diazo-	0.18
38	Naphthalene, 1,1'-(1,2-ethanediyl)bis[decahydro-	0.08
39	2-(4a,8-Dimethyl-1,2,3,4,4a,5,6,7-octahydro-naphthalen-2-yl)-prop-2-en-1-ol	0.15
40	Naphthalene, 1,2,3,5,6,7,8,8a-octahydro-1,8a-dimethyl-7-(1-methylethenyl)-, [1S-(1.alpha.,7.alpha.,8a.alpha.)]-	0.84
41	3-Oxatricyclo[20.8.0.0(7,16)]triaconta-1(22),7(16),9,13,23,29-hexaene	0.26
42	2(3H)-Benzofuranone, 6-ethenylhexahydro-3,6-dimethyl-7-(1-methylethenyl)-, [3S-(3.alpha.,3a.alpha.,6.alpha.,7.beta.,7a.beta.)]-	0.36
43	Caryophyllene	0.09
44	(-)-Isoaromadendrene-(V)	4.04
45	Azulene, 1,2,3,5,6,7,8,8a-octahydro-1,4-dimethyl-7-(1-methylethenyl)-, [1S-(1.alpha.,7.alpha.,8a.beta.)]-	0.84
46	(-)-Spathulenol	0.38
47	9.beta.-Acetoxy-3,5.alpha.,8-trimethyltricyclo[6.3.1.0(1,5)]dodec-3-ene	0.16
48	Tricyclo[4.2.2.0(2,5)]dec-7-ene, 7-(5-hexynyl)-	2.52
49	Eudesma-5,11(13)-dien-8,12-olide	0.89

50	Bicyclo[5.3.0]decane, 2-methylene-5-(1-methylvinyl)-8-methyl-	1.10
51	Azuleno[4,5-b]furan-2(3H)-one, 3a,4,6a,7,8,9,9a,9b-octahydro-6-methyl-3,9-bis(methylene)-, [3aS-(3a.alpha.,6a.alpha.,9a.alpha.,	13.23
52	.tau.-Cadinol	0.39
53	Kauran-18-al, 17-(acetyloxy)-, (4.beta.)-	1.39
54	2-Butenoic acid, 2-methyl-, 2-(acetyloxy)-1,1a,2,3,4,6,7,10,11,11a-decahydro-7,10-dihydroxy-1,1,3,6,9-pentamethyl-4a,7a-epoxy-5	0.25
55	13-Docosenamide, (Z)-	0.40
56	9-Methyl-10,12-hexadecadien-1-ol acetate	0.37
57	Cyclohexanol, 2-methyl-3-(1-methylethenyl)-, (1.alpha.,2.alpha.,3.alpha.)-	0.21
58	Piperine	0.51
59	6-epi-shyobunol	0.22
60	(R)-(-)-14-Methyl-8-hexadecyn-1-ol	0.88
61	5.alpha.-Hydroxy-4.alpha.,8,10,11-tetramethyltricyclo[6.3.0.0(2,4)]undec-10-ene	0.54
62	Card-20(22)-enolide, 3,5,14,19-tetrahydroxy-, (3.beta.,5.beta.)-	0.57
63	Ergost-5-en-3-ol, (3.beta.)-	0.26
64	Stigmasterol	0.44
65	9.beta.-Acetoxy-3.beta.-hydroxy-3,5.alpha.,8-trimethyltricyclo[6.3.1.0(1,5)]dodecane	1.45
66	.beta.-Sitosterol	1.14
67	geranyl-.alpha.-terpinene	0.85
68	9,19-Cycloergost-24(28)-en-3-ol, 4,14-dimethyl-, acetate, (3.beta.,4.alpha.,5.alpha.)-	3.51
69	Tricyclo[20.8.0.0(7,16)]triacontane, 1(22),7(16)-diepoxy-	5.42
